# On the use of solid ^133^Ba sources as surrogate for liquid ^131^I in SPECT/CT calibration: a European multi-centre evaluation

**DOI:** 10.1186/s40658-023-00582-3

**Published:** 2023-11-23

**Authors:** Johannes Tran-Gia, Ana M. Denis-Bacelar, Kelley M. Ferreira, Andrew P. Robinson, Christophe Bobin, Lara M. Bonney, Nicholas Calvert, Sean M. Collins, Andrew J. Fenwick, Domenico Finocchiaro, Federica Fioroni, Katerina Giannopoulou, Elisa Grassi, Warda Heetun, Stephanie J. Jewitt, Maria Kotzasarlidou, Michael Ljungberg, Valérie Lourenço, Daniel R. McGowan, Jamie Mewburn-Crook, Benoit Sabot, James Scuffham, Katarina Sjögreen Gleisner, Jaroslav Solc, Cheick Thiam, Jill Tipping, Jill Wevrett, The MRT Dosimetry Collaboration, Michael Lassmann

**Affiliations:** 1 Department of Nuclear Medicine, University Hospital Würzburg, Oberdürrbacher Str. 6, 97080 Würzburg, Germany; 2 National Physical Laboratory, Hampton Road, Teddington, UK; 3 Université Paris-Saclay, CEA, List, Laboratoire National Henri Becquerel (LNE-LNHB), 91120 Palaiseau, France; 4 Department of Medical Physics and Clinical Engineering, Churchill Hospital, Oxford University Hospitals NHS Foundation Trust, Oxford, UK; 5 Christie Medical Physics and Engineering (CMPE), The Christie NHS Foundation Trust, Manchester, UK; 6 School of Mathematics and Physics, University of Surrey, Guildford, UK; 7 Medical Physics Unit, Azienda USL-IRCCS Di Reggio Emilia, Reggio Emilia, Italy; 8 Nuclear Medicine Department, “THEAGENIO” Anticancer Hospital, Thessaloniki, Greece; 9 Medical Radiation Physics, Lund, Lund University, Lund, Sweden; 10 Department of Oncology, University of Oxford, Oxford, UK; 11 Royal Surrey County Hospital, Royal Surrey NHS Foundation Trust, Guildford, UK; 12 Czech Metrology Institute, Okruzni 31, 638 00 Brno, Czech Republic; 13 Medical Physics Unit, Azienda Ospedaliero-Universitaria Policlinico di Modena, Modena, Italy

**Keywords:** ^133^Ba, Barium-133, ^131^I, Radioiodine, Solid surrogate source, Quantitative SPECT/CT, Comparison exercise, Multi-centre, Calibration

## Abstract

**Introduction:**

Commissioning, calibration, and quality control procedures for nuclear medicine imaging systems are typically performed using hollow containers filled with radionuclide solutions. This leads to multiple sources of uncertainty, many of which can be overcome by using traceable, sealed, long-lived surrogate sources containing a radionuclide of comparable energies and emission probabilities. This study presents the results of a quantitative SPECT/CT imaging comparison exercise performed within the MRTDosimetry consortium to assess the feasibility of using ^133^Ba as a surrogate for ^131^I imaging.

**Materials and methods:**

Two sets of four traceable ^133^Ba sources were produced at two National Metrology Institutes and encapsulated in 3D-printed cylinders (volume range 1.68–107.4 mL). Corresponding hollow cylinders to be filled with liquid ^131^I and a mounting baseplate for repeatable positioning within a Jaszczak phantom were also produced. A quantitative SPECT/CT imaging comparison exercise was conducted between seven members of the consortium (eight SPECT/CT systems from two major vendors) based on a standardised protocol. Each site had to perform three measurements with the two sets of ^133^Ba sources and liquid ^131^I.

**Results:**

As anticipated, the ^131^I pseudo-image calibration factors (cps/MBq) were higher than those for ^133^Ba for all reconstructions and systems. A site-specific cross-calibration reduced the performance differences between both radionuclides with respect to a cross-calibration based on the ratio of emission probabilities from a median of 12–1.5%. The site-specific cross-calibration method also showed agreement between ^133^Ba and ^131^I for all cylinder volumes, which highlights the potential use of ^133^Ba sources to calculate recovery coefficients for partial volume correction.

**Conclusion:**

This comparison exercise demonstrated that traceable solid ^133^Ba sources can be used as surrogate for liquid ^131^I imaging. The use of solid surrogate sources could solve the radiation protection problem inherent in the preparation of phantoms with ^131^I liquid activity solutions as well as reduce the measurement uncertainties in the activity. This is particularly relevant for stability measurements, which have to be carried out at regular intervals.

**Supplementary Information:**

The online version contains supplementary material available at 10.1186/s40658-023-00582-3.

## Introduction

There is a growing interest in the use of quantitative single-photon emission computed tomography and X-ray computed tomography (SPECT/CT) imaging, driven by a surge in theranostics and the need to optimise the absorbed doses delivered in molecular radiotherapy [1]. Absolute quantification in SPECT/CT imaging enables the direct evaluation of the activity concentration within a given volume of tissue, where the number of counts in each voxel is proportional to the activity. Although the individual steps for calibrating SPECT systems are well described [2], there are many sources of uncertainties associated with this process, including phantom preparation and radionuclide calibrator measurements, as well as those associated with reconstruction, image correction methods and post-processing of the images.

Radioiodine (^131^I) is still one of the most commonly used radionuclides in molecular radiotherapy, which is mainly used for the treatment of benign and malignant thyroid disorders [3]. Quantitative imaging for dosimetry of ^131^I-based radiopharmaceuticals with a marketing authorisation is applied in several clinical use cases. This comprises the treatment of benign thyroid diseases [4, 5], thyroid cancer [6, 7] with Na[^131^I]I or neuroendocrine tumours with [^131^I]mIBG. [8, 9].

^133^Ba has been previously proposed as a potential surrogate for the calibration of ^131^I imaging due to its longer half-life and similarity in the energy of its most abundant gamma-ray at 356 keV, as compared to 364 keV for ^131^I. Zimmerman et al. assessed activity quantification using planar, SPECT, and SPECT/CT imaging in an international multi-centre study using traceable ^133^Ba sources as a surrogate for ^131^I [10]. Based on data from the participants in nine countries, the authors concluded that solid surrogate sources could help in avoiding inherent problems with on-site activity measurements and phantom preparation in multi-centre studies. Moreover, large uncertainties for planar and SPECT imaging led to the conclusion that SPECT/CT was the preferred method. They also demonstrated the need for training and standardised acquisition and processing protocols to achieve accurate and reproducible activity quantification.

In the light of this knowledge, the EMPIR MRTDosimetry project, formed by a European collaboration between metrologists and nuclear medicine researchers, undertook the development and testing of traceable ^133^Ba sources that could replace liquid ^131^I for commissioning, calibration and quality control of quantitative SPECT/CT imaging. This publication presents the design and traceable production of two sets of ^133^Ba cylinders at two National Metrology Institutes, the standard operating procedure (SOP) for quantitative SPECT/CT imaging, and the results from a comparison exercise performed within seven members of the consortium with access to eight SPECT/CT systems. To improve reproducibility of future studies, the SOP as well as the source designs has been made available in an open data repository [11].

## Methods

### Participants and equipment

Seven members of the MRTDosimetry consortium participated in the comparison exercise (Azienda USL-IRCCS di Reggio Emilia, The Christie NHS Foundation Trust, Lund University, Oxford University Hospitals NHS Foundation Trust, Royal Surrey NHS Foundation Trust, “THEAGENIO” Anticancer Hospital, and University Hospital Würzburg). The participants were required to have access to a SPECT/CT system with high-energy collimators as well as methods for attenuation and scatter correction. In total, eight SPECT/CT imaging systems (5 × General Electric (GE) and 3 × Siemens) were included in the study. The individual setup details are given in Table 1, where four different combinations of reconstruction and correction methods were performed with system 2 (setups S2a-S2d). The description of the acquisition and reconstruction settings are provided in later sections.

**Table 1:** SPECT/CT systems and setups used in the comparison exercise

Setup	Vendor	Model	#CT rows	Crystal Thickness	Reconstruction software	Scatter correction	Resolution recovery
S1	Siemens	Symbia T2	2	9.5 mm	Siemens e-soft	TEW^a^	Yes
S2a	General Electric	Discovery 670	16	9.5 mm	GE Xeleris	TEW^a^	Yes
S2b	General Electric	Discovery 670	16	9.5 mm	Hermes	Monte Carlo^b^	Yes (Monte Carlo)
S2c	General Electric	Discovery 670	16	9.5 mm	Hermes	Monte Carlo^b^	Yes
S2d	General Electric	Discovery 670	16	9.5 mm	Hermes	TEW^a^	Yes
S3	General Electric	Discovery 670	16	15.9 mm	In-house	ESSE^c^	Yes
S4	General Electric	Discovery 670	16	9.5 mm	GE Xeleris	TEW^a^	Yes
S5	General Electric	Optima NM/CT 640	4	9.5 mm	GE Xeleris	TEW^a^	Yes
S6	General Electric	Optima NM/CT 640	4	9.5 mm	Hermes	TEW^a^	Yes
S7	Siemens	Symbia Intevo Bold	16	9.5 mm	Siemens e-soft	TEW^a^	Yes
S8	Siemens	Symbia T2	2	15.9 mm	Siemens e-soft	TEW^a^	Yes

^a^TEW, triple-energy window scatter correction [12]

^b^Monte Carlo-based scatter correction [13]

^c^ESSE, effective scatter source estimation [14]

### Phantom design

SPECT/CT activity calibration factors are often determined in large-volume phantoms to reduce the influence of the partial volume effect. However, the production of such a large cylinder uniformly filled with a sufficiently high activity concentration of solidified ^133^Ba is challenging. Therefore, the comparison exercise was performed based on a set of four smaller cylinders of different sizes, similar to those proposed by Zimmerman et al. [10]. Although it was not the primary aim of this study, the resulting multi-centre dataset was used to provide a comparison of the relative impact of spatial resolution and partial volume effects between ^131^I and ^133^Ba within the cross-comparison.

**Fig. 1:**
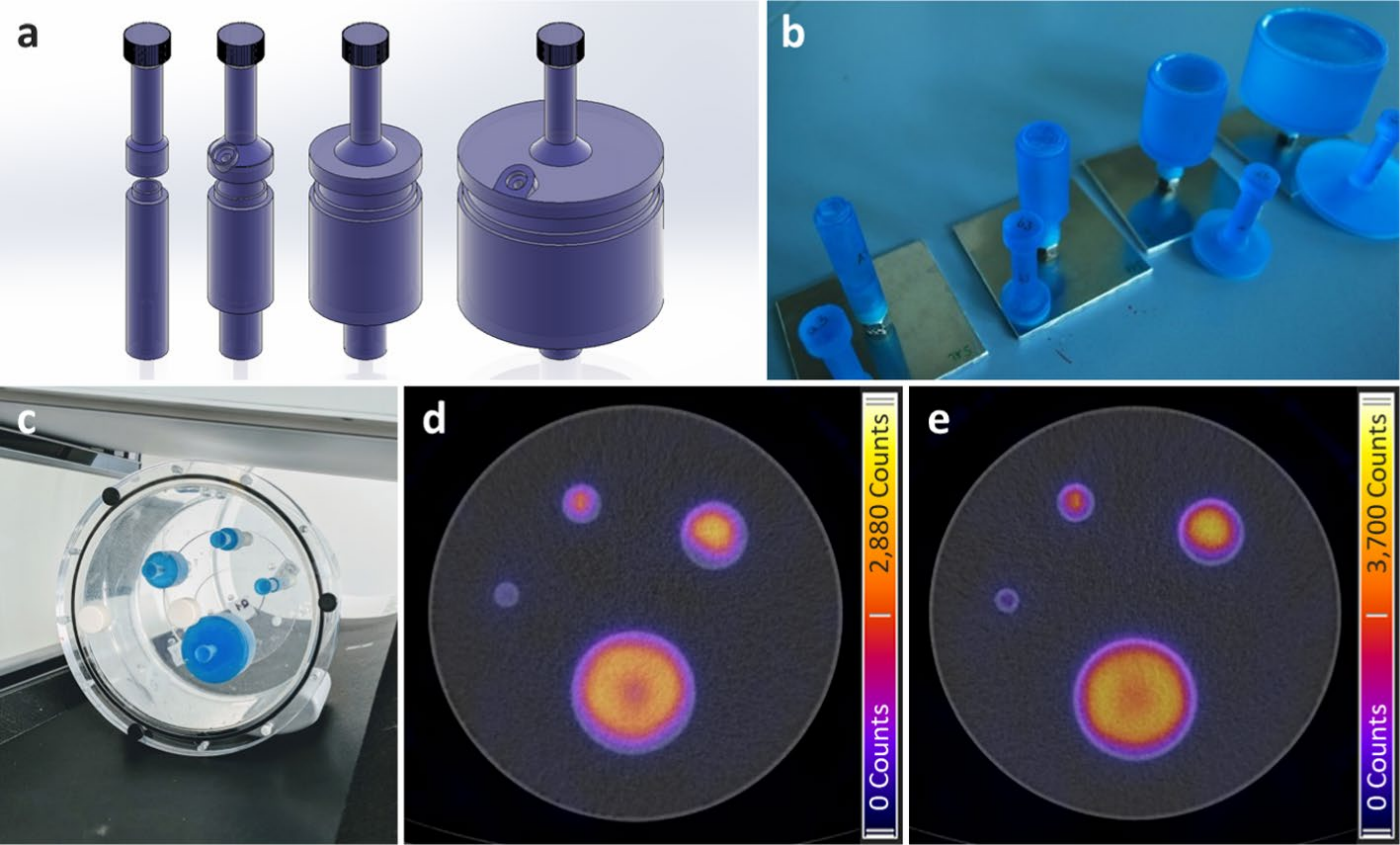
**a** CAD models of the solid and fillable cylinders including cap, **b** production of the solid sources at CEA, (c) SPECT/CT measurement of the four cylinders mounted in a Jaszczak cylindrical phantom, (d) SPECT/CT fusion of the CEA ^133^Ba sources measured with setup S7, (e) SPECT/CT fusion of the liquid ^131^I sources measured with setup S7

Computer-aided designs (CADs) for cylinders with four active volumes (Table 2) were produced (Fig. 1a). A wall thickness of 3 mm was used, with no difference in attenuation expected due to the nearly equivalent attenuation of water and resin in the expected energy range (0.078% difference in attenuation for 344 keV [15]). To enable an evaluation of the partial volume effect, different height diameter ratios at a constant height were used. Two versions of cylinder caps were designed, one for containing a resin mixed with ^133^Ba and one for injection of a solution with ^131^I. A thread was glued to the bottom of the cylinders to enable mounting the cylinders to a custom-made baseplate using double-threaded plastic rods. Cylinders and caps were produced using a stereolithography (SLA) 3D printing system (Formlabs Form 2) using the Formlabs Tough (v5) photopolymer resin formulation (density when cured = (1.15–1.20) g·cm^−3^), resulting in a durable and partially transparent model (a useful feature for judging the level when filling). Threads (M3) were added to the injection caps before being fixed into position using epoxy resin and tested to ensure the resulting models were watertight. The designs can be downloaded from the MRTDosimetry data repository [11].

**Table 2:** Geometries and activities for the cylindrical solid ^133^Ba sources produced within this project at Laboratoire National Henri Becquerel (CEA) and the Czech Metrology Institute (CMI). Reference date (UTC): 15 December 2018 (12:00). All uncertainties are expanded uncertainties (*k* = 2)

ID	Diameter (mm)	Height (mm)	Volume (cm^3^)	CMI		CEA	
				Activity (kBq)	Active volume (cm^3^)	Activity (kBq)	Active volume (cm^3^)
C1	7.5	38.0	1.68	155.8 ± 3.4	1.62 ± 0.02	333.5 ± 8.7	1.66 ± 0.03
C2	15.0	38.0	6.72	649 ± 14	6.76 ± 0.07	1343 ± 35	6.67 ± 0.13
C3	30.0	38.0	26.9	2583 ± 57	26.9 ± 0.3	5150 ± 130	25.6 ± 0.5
C4	60.0	38.0	107.4	10,304 ± 227	107.3 ± 1.2	20,350 ± 530	101.1 ± 2.0

To optimise the placement of the sources in a standard Jaszczak phantom (cylinder with a fillable volume of 21.6 cm diameter and 18.6 cm height), the collimator-dependent spill-out of counts was estimated by the convolution of a Gaussian function with 20 mm full width at half maximum. This value was chosen to provide a representative worst-case scenario of the reconstructed spatial resolution for ^131^I based on previous experience with the calibration of SPECT/CT systems with radioiodine in a clinical setting, but was not specifically measured in this study. A laser-cut baseplate for attachment of the sources was produced according to this optimal positioning. For a SPECT/CT measurement, the baseplate and the support rods were mounted in the Jaszczak cylinder, four cylinders (either one of two sets of ^133^Ba solid sources or liquid ^131^I sources) were attached to the mounting baseplate, and the Jaszczak cylinder was filled with water.

### Solid ^133^Ba source production

Two sets of ^133^Ba cylinders (four inserts each) were produced, one at the Laboratoire National Henri Becquerel (CEA) and one at the Czech Metrology Institute (CMI).

#### Commissariat à l'énergie atomique et aux énergies alternatives (CEA) sources

At CEA, a two-component epoxy resin (Stycast 1264, [16]) was spiked with ^133^Ba. The amine component was mixed with a limited amount of radioactive aqueous phase, and after mixing with the epoxy component for 30 min, the spiked resin was cured at room temperature (~ 48 h total solidification time). The resulting spiked resin has a density of (1.140 ± 0.011) g·cm^−3^ (*k* = 1). The uniformity of the 200 mL batch was assessed by measuring eighteen 2.5 mL subsamples by 4π gamma counting, aliquoted throughout the whole filling process of the four geometries. The dispersion was below 0.1%. The activity of the resin measured by gamma spectrometry for each geometry was in agreement with the spiking value derived from weighing. The relative combined standard uncertainty on the activity in each cylinder is 1.3%, which is below the 2.0% target limit. Following ISO 9978 standard, leakage and contamination tests were conducted: wipe test on the spiked resin, wipe test on the closed vessels, and an immersion test. For the wipe tests, the detection limit reached was below 1 Bq and no activity was detected. For the immersion test, a closed geometry filled with 1 MBq of ^133^Ba spiked resin was immersed in water at room temperature for up to 4 days. The water was measured by gamma spectrometry, and no activity was measured (detection limit of 0.21 Bq). Figure 1b shows the unfinished sources during production.

#### Czech metrology institute (CMI) sources

CMI developed a set of reference ^133^Ba sources with the radionuclide fixed in two-component silicone rubber Lukopren (Lučební závody Kolín, Czech Republic). Drops of ^133^Ba water solution were added into the liquid rubber and stirred well. Pouring the second component of the rubber resulted in solidification of the solution within a few tens of minutes, fixing the radionuclide in the matrix. Distribution of the radionuclide was uniform within ± 1% in the whole volume of the solidified source. As with the CEA sources, an immersion test according to ISO9978, in Sect. 5.1.4, was applied to measure the radioactivity leakage from the manufactured encapsulated sources. Results of measurement with liquid scintillation analyser Tri-Carb 2910TR (PerkinElmer, USA) were below the detection limit of 1 Bq for all four sources. The relative combined standard uncertainty of the activity of the manufactured sources reached 1.1% and consisted of the uncertainty of the silicone rubber density (1.0%), activity of ^133^Ba stock solution (0.4%), and weighting (0.06%).

### Solid ^133^Ba phantom preparation

The two sets of sources were sequentially distributed to the participating centres during the comparison exercise. In addition, each participant received a mounting baseplate, support rods, a set of screws, and a set of empty 3D-printed cylinders to be filled with the ^131^I solution.

### Liquid ^131^I phantom preparation

Two phantoms containing ^131^I were prepared at each site: a uniformly filled Jaszczak cylindrical phantom to assess the setup-specific image calibration factor (ICF) and a phantom containing the set of four 3D-printed fillable cylinders to assess the partial volume effect.

The ^131^I activity for the ICF assessment with the Jaszczak phantom was measured in the local radionuclide calibrator before and after injection into the phantom using a traceable ^131^I calibration factor (dial setting specific to the radionuclide, measurement container, measurement geometry inside the calibrator, and filling level). To ensure a uniform activity distribution of the volatile ^131^I in the phantom, a carrier solution of sodium hydroxide (0.1 mol dm^−3^) with inactive iodine (10 μg g^−1^) was used. The activities of the ICF measurement at the time of SPECT scanning had a median value of 39.9 MBq (range 34.2–86.9 MBq).

For the four fillable cylinders, a stock solution was prepared at each site by weighing a container before and after adding 160 mL of sodium hydroxide (0.1 mol dm^−3^) with inactive iodine (10 μg g^−1^) as carrier solution as well as ~ 30 MBq of liquid ^131^I (from traceable activity measurement as described for the ICF measurement). The activity concentration was calculated as the ratio of dispensed activity to volume. The cylinders were filled by weighing each empty cylinder separately, injecting the stock solution, and re-weighing the filled cylinder. The activity inside each cylinder was then calculated as the product of activity concentration and active volume. The total ^131^I activity concentrations at the time of SPECT scanning had a median of 0.18 MBq·mL^−1^ (range 0.16–0.21 MBq mL^−1^) corresponding to total activities of 0.30, 1.2, 4.9, and 19 MBq in the cylinders with 7.5 (C1), 15 (C2), 30 (C3), and 60 (C4) mm diameter, respectively. As with the ^133^Ba cylinders, the ^131^I cylinders were attached to the mounting baseplate with the rods, and the phantom was filled with water.

### Data acquisition and reconstruction

The measurements included the ICF determination with the Jaszczak phantom and separate measurements of the three sets of four small cylinders (CEA ^133^Ba, CMI ^133^Ba, and liquid ^131^I) mounted in the water filled Jaszczak phantom. All measurements within the scope of the exercise were performed according to a dedicated SOP, containing information on the required equipment, instructions on energy peak alignment (mandatory), uniformity quality control (optional), phantom filling and positioning, SPECT/CT acquisition parameters, reconstruction and correction methods, delineation of volumes of interest (VOI), and file transfer.

SPECT/CT imaging of the phantoms was performed according to the acquisition parameters given in Table 3. The acquisitions were performed with the phantom (placed with the largest cylinder phantom insert closest to the patient bed) oriented axially using a high-energy collimator and with a standard low-dose CT protocol for attenuation correction. An example of the fully assembled phantom and its positioning on a SPECT/CT system can be seen in Fig. 1c. For ^133^Ba acquisitions, an energy peak alignment was performed using the smallest ^133^Ba source. A 60-min acquisition was performed for the ^131^I ICF measurement, while 30-min acquisitions were performed for all cylinder measurements (CEA and CMI ^133^Ba, and ^131^I). The images were reconstructed using an ordered subset expectation maximisation (OSEM) iterative reconstruction with 30 iterations and 2 subsets without post-filtering [17]. Convergence had previously been verified at a representative site. The use of triple energy window (TEW) scatter correction and resolution recovery methods was recommended. However, the comparison exercise also included participants with small differences in the reconstruction software and corrections applied, as in the case of participant 3, which used in-house reconstruction software and the ESSE scatter correction. Details on the reconstruction software and scatter correction method used by each participant are shown in Table 1. Examples of SPECT/CT reconstructions of solid CEA ^133^Ba sources and ^131^I-filled cylinders are given in Figs. 1d and e, respectively.

**Table 3:** SPECT/CT acquisition parameters

Radionuclide	^133^Ba cylinders	^131^I cylinders	^131^I uniform Jaszczak
Collimator	High-energy		
Number of energy windows	3		
Photopeak energy (keV)	356.0 ± 26.7 (15% width)	364.5 ± 36.6 (20% width)	364.5 ± 36.6 (20% width)
Lower scatter energy (keV)	321.0 ± 8.0 (5% width)	317.1 ± 9.5 (6% width)	317.1 ± 9.5 (6% width)
High scatter energy (keV)	403.0 ± 20.2 (10% width)	411.9 ± 12.4 (6% width)	411.9 ± 12.4 (6% width)
Flood uniformity	As per clinical imaging protocol for ^131^I		
Matrix	128		
SPECT orbit	Body contour		
Number of projections	120 (60 per detector)		
Time/projection	30 s	30 s	60 s
Detector movement	Step-and-shoot		
CT	Standard low-dose protocol		

### Data analysis

#### Analysis of the solid ^133^Ba and the liquid ^131^I measurements

First, the ICF for ^131^I was calculated based on the SPECT images of the Jaszczak cylinder as:

1 $$ICF=\frac{{C}_{Jasz}}{{T}_{Jasz}\cdot {A}_{Jasz}}$$

Here, ${C}_{Jasz}$ is the number of counts in an enlarged CT-based VOI placed around the uniformly filled Jaszczak cylindrical phantom to account for partial volume effect, ${T}_{Jasz}$ is the acquisition time duration, and ${A}_{Jasz}$ is the activity in the phantom as measured in the radionuclide calibrator and decay corrected to the time of acquisition.

For each of the cylinder inserts (^131^I and ^133^Ba), a pseudo-ICF value, by which ICF values that are influenced by partial volume effects (reduced ICF due to spill-out) will be described hereafter, was calculated as:

2 $$Pseudo{\text{-}}ICF=\frac{{C}_{cyl}}{{T}_{cyl}\cdot {A}_{cyl}}$$

Here, ${C}_{cyl}$ is the number of counts in the cylinder VOI, ${T}_{cyl}$ is the acquisition time duration, and ${A}_{cyl}$ is the activity in the phantom decay corrected to the time of acquisition. The choice of VOI drawing technique was left to the participating sites to reflect the heterogeneity in clinical workflows. However, care was taken in the evaluation to only compare VOIs whose volumes matched the cylinder volumes. While one site chose to draw the VOIs based on the nominal cylinder dimensions (S1), all other sites used a thresholding method to match the physical active volume of the given cylinder (S2-S8).

The combined standard uncertainty in the ICF and pseudo-ICF values was calculated as the square root of the sum of the squared standard uncertainty components, including counts, time, and activity. As the study design required on-site reconstruction, projection data were not available, and therefore, the uncertainty in the counts within the considered volume was assumed to follow Poisson statistics and calculated as the square root of the number of counts [18]. A one-second uncertainty in scan duration was assumed for all the scans acquired. The uncertainty on the radionuclide calibrator measurements included the uncertainty on the calibration setting, reproducibility, linearity, uncertainty due to background correction, uncertainty associated with decay correction, and statistical uncertainty. The sources of uncertainty related to the measurement on the radionuclide calibrators were considered to follow a normal distribution. The weighing uncertainty was also considered for each cylinder, and a rectangular distribution was assumed. The contribution to the standard uncertainties was combined in quadrature to estimate the combined standard uncertainty of the activity dispensed to the cylinders.

The differences between the pseudo-ICFs obtained with the CEA and CMI ^133^Ba sources were assessed for statistical significance using a nonparametric two-sided Wilcoxon signed-rank test under the null hypothesis of zero median for the differences between the paired CEA and CMI pseudo-ICF values.

#### Cross-calibration between ^133^Ba and ^131^I

The relationship between the pseudo-ICFs for liquid ^131^I and solid ^133^Ba was studied based on a setup-specific cross-calibration line, which relates the ^131^I-based counts measured on a specific system for the cylinder geometry of different sizes to the ^133^Ba-based counterparts. Due to setup- as well as radionuclide-specific differences, the linear relationship differs from a cross-calibration based on only the ratio of ^133^Ba and ^131^I emission intensities. To test the performance of an experimental site-specific cross-calibration, the correlation between ^133^Ba and ^131^I pseudo-ICFs for each setup was determined by fitting a non-weighted linear model. The relative percentage change between ^131^I and ^133^Ba, corrected with both setup-specific and emission probability-based cross-calibrations, were calculated to assess the performance of both cross-calibration methods. In addition, to assess the potential use of ^133^Ba sources to calculate a volume-dependent partial volume correction, pseudo-recovery curves (pseudo-ICF against volume) were determined for ^131^I, uncorrected ^133^Ba, and ^133^Ba corrected with both cross-calibration methods by fitting a non-weighted non-linear model.

## Results

A representative example of the design and fabrication of the cylindrical sources as well as SPECT/CT measurements and reconstructions of the physical phantom with the cylindrical sources are shown in Fig. 1.

### Solid ^133^Ba measurements

The Wilcoxon test for the two ^133^Ba sets of sources showed no evidence to reject the null hypothesis of zero median difference between the CMI and CEA sources at a 5% confidence level (P = 0.32). Therefore, for the rest of the analysis, the two ^133^Ba measurements were combined using the average value. For completeness, a plot of the individual ^133^Ba cylinder pseudo-ICFs for the CEA and CMI sources as well as a table with the numerical values can be found in Additional file 1. The uncertainty in the averaged pseudo-ICF ($\overline{Pseudo{\text{-}}ICF}$) includes two components combined in quadrature: the uncertainty of the average calculated from the propagation of uncertainties of the two uncorrelated measurements and an additional uncertainty to account for the deviation between the two measurements assuming a uniform distribution [19]:

3 $${u\left({\overline{Pseudo{\text{-}}ICF}}\right)}=\sqrt{\begin{array}{c}\frac{{u\left({Pseudo\text{-}ICF}_{CEA}\right)}^{2}+{u\left({Pseudo\text{-}ICF}_{CMI}\right)}^{2}}{4}\\ +{\frac{\left({Pseudo\text{-}ICF}_{CEA}-{Pseudo\text{-}ICF}_{CMI}\right)}{12}}^{2}\end{array}}$$

**Fig. 2:**
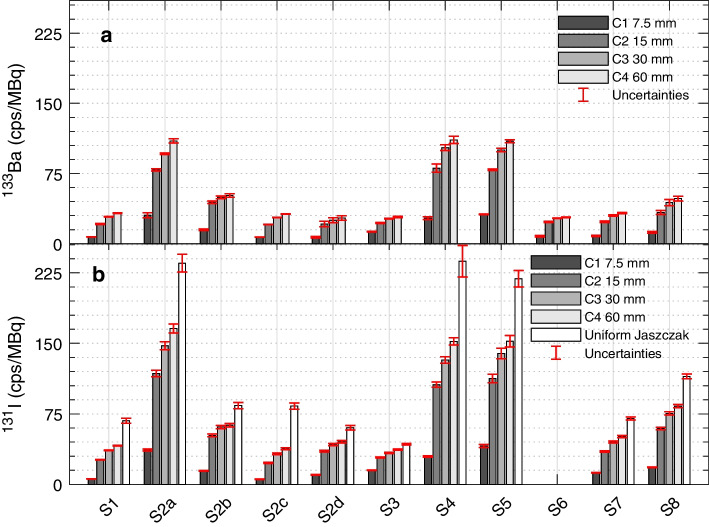
**a** ^133^Ba-cylinder combined average pseudo-ICFs, **b** ^131^I cylinder pseudo-ICFs and ICF from the uniformly filled Jaszczak phantom measurement. Note that both data sets are shown in the same y-axis scale. A plot of the individual ^133^Ba cylinder pseudo-ICFs for the CEA and CMI sources and a table with the numerical values can be found in Additional file 1

The individual CEA and CMI pseudo-ICFs as well as their combined average are provided as supplementary material. The averaged pseudo-ICFs for the ^133^Ba cylinders are shown in Fig. 2a. All Siemens systems (S1, S7, and S8) show similar pseudo-ICFs, with a slight increase for S8, which has a thicker crystal and therefore a higher sensitivity. GE systems using the vendor reconstruction software (S2a, S4, and S5) also have comparable ICFs and have the highest pseudo-ICFs of all setups. GE acquisitions reconstructed using the vendor neutral Hermes (Hermes Medical Solutions, Stockholm, Sweden) (S2a-d and S6) and in-house software (S3) show lower ICFs than the GE reconstruction. Of these, S2b, which uses Hermes Monte Carlo-based scatter and resolution recovery methods, shows an increase in ICF as compared to the other Hermes-based reconstructions. The larger uncertainties observed in some of the cylinders, e.g. C2-C4 for S4, are due to a larger deviation between the two measurements. As expected, a reduction in pseudo-ICF values with decreasing volumes was observed for all setups due to an increase in partial volume-related spill-out of counts.

### Liquid ^131^I measurements

The pseudo-ICF values based on the small cylinders and the ICF values based on the large-volume Jaszczak phantom for ^131^I are shown in Fig. 2b. During the data analysis, an inconsistency was observed in the ^131^I data reported by S6; therefore, the data of this site have not been used in the rest of the comparison between ^133^Ba and ^131^I.

As with the ^133^Ba measurements, the ICF and pseudo-ICF values vary depending on the system vendor and setup, showing similar variations as ^133^Ba for the different reconstruction and correction methods. Again, a partial volume-related reduction in pseudo-ICF values with decreasing volumes was observed.

### Comparison between ^133^Ba and ^131^I

**Fig. 3:**
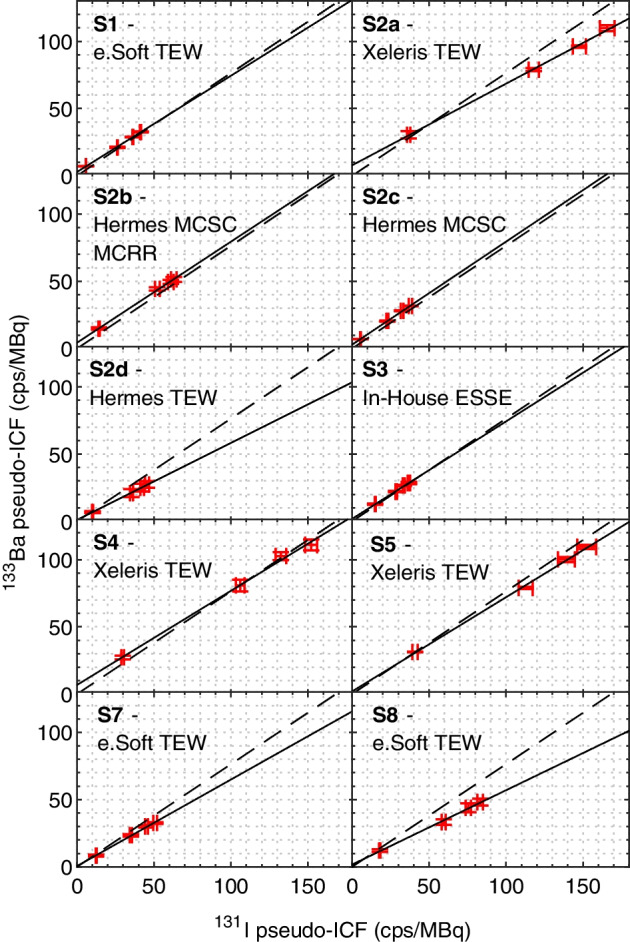
Setup-specific cross-calibration between ^133^Ba and ^131^I. The solid line represents the setup-specific cross-calibration line (linear model fit, see Table 4), and the dashed line represents the ratio of the emission probabilities between ^133^Ba and ^131^I. Note that the ^133^Ba pseudo-ICF values represent the combined average between both sources

The setup-specific cross-calibration lines (i.e. the relationship between the pseudo-ICF values of ^131^I and ^133^Ba) are shown in Fig. 3. In addition, the ratio between the gamma-ray emission probabilities (0.764 ± 0.005) of the 356 keV ^133^Ba energy peak (62.05 ± 0.19) % and the 364 keV ^131^I energy peak (81.2 ± 0.5) % is displayed for reference [20, 21]. The corresponding fit parameters are given in Table 4.

**Table 4:** Fitted parameters (offset and slope) of the setup-specific cross-calibration line for each individual setup, with their corresponding standard uncertainties determined from the linear fit. The theoretical ratio of gamma-ray emission probabilities of ^133^Ba and ^131^I is 0.764 ± 0.005

Cross-calibration linear model parameters		
Setup	Offset	Slope
S1	2.80 ± 0.43	0.716 ± 0.014
S2a	7.8 ± 1.8	0.607 ± 0.014
S2b	4.30 ± 0.93	0.752 ± 0.018
S2c	2.88 ± 0.30	0.766 ± 0.011
S2d	1.00 ± 0.50	0.573 ± 0.014
S3	1.84 ± 0.90	0.725 ± 0.030
S4	6.8 ± 3.5	0.701 ± 0.030
S5	2.0 ± 2.2	0.701 ± 0.018
S7	0.57 ± 0.85	0.644 ± 0.022
S8	1.7 ± 1.3	0.553 ± 0.021

As has been pointed out in 2000 by Dewaraja et al. [22], septal penetration by high-energy photons of ^131^I (723 keV, 637 keV) contributes substantially to the background. The conventional TEW technique for background correction might thus lead to a difference between true scatter and the TEW estimate in the order of 10–20%. As a consequence, this effect would slightly lower the value of the correction factor, which explains the mainly lower slope of the correction line compared to the theoretical slope.

**Fig. 4:**
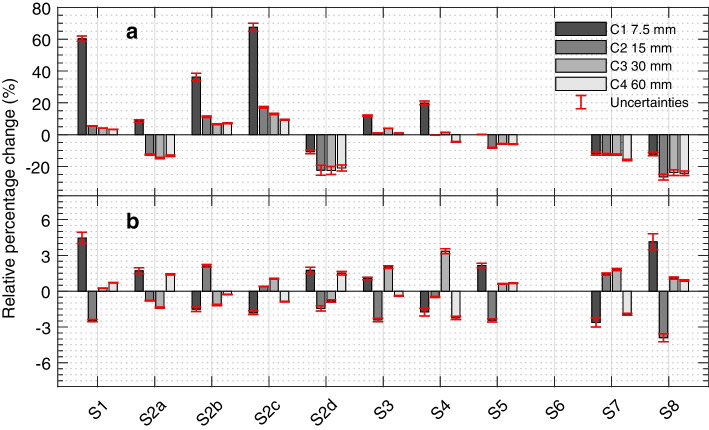
Relative percentage change between ^133^Ba and ^131^I using the emission probability-based cross-calibration factor (**a**) and the experimental setup-specific cross-calibration line (**b**). Note the two different scales in the y-axes

The relative differences between the ^131^I and ^133^Ba pseudo-ICFs cross-calibrated with the setup-specific cross-calibration line and emission probability-based ratio are shown in Fig. 4. The median [range] absolute relative difference across setups is reduced from 12 [0.065–67] % to 1.5 [0.25–4.5] % when the site-specific cross-calibration method is used, with the largest improvements observed in the smallest cylinders.

**Fig. 5:**
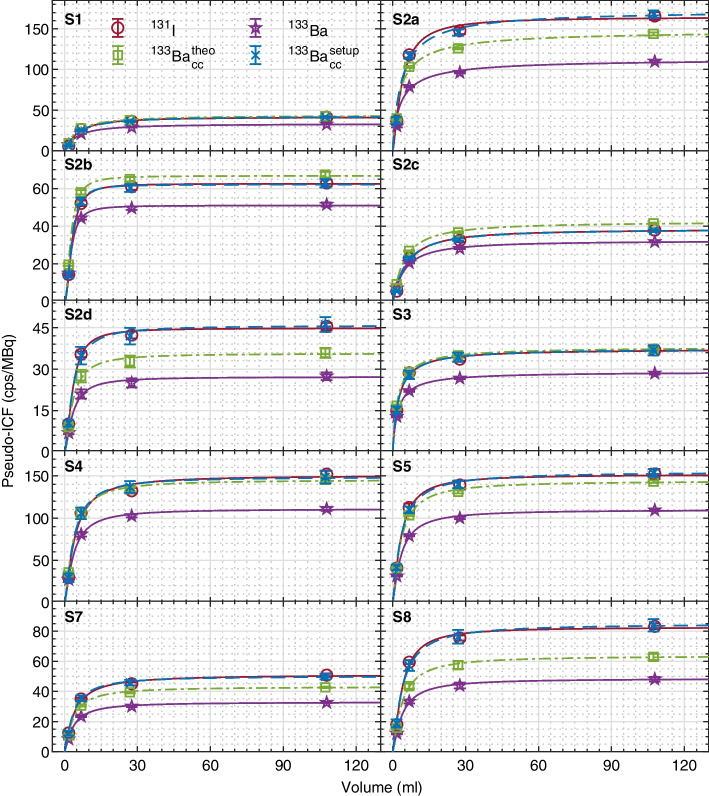
Pseudo-recovery curves (ICF values in cps/MBq as a function of cylinder volumes) for: ^131^I (red circle), ^133^Ba (purple star), ^133^Ba cross-calibrated (cc) using the theoretical emission probabilities ratio (green square), and ^133^Ba cross-calibrated using the experimentally measured setup-specific factors from Table 4 (blue cross). Note that each pair of subplots in a row has different scales in the y-axes

The pseudo-recovery curves for ^131^I, ^133^Ba, ^133^Ba cross-calibrated using the theoretical ratio of emission probabilities, and ^133^Ba cross-calibrated using the setup-specific cross-calibration line from Table 4 are shown in Fig. 5. A good agreement between the ^131^I and ^133^Ba curves is shown for all sites when using the setup-specific cross-calibration line, highlighting the potential use of ^133^Ba sources to determine recovery coefficients to correct for partial volume effects.

## Discussion

In this study, the experimental setup-specific cross-calibration between ^131^I and ^133^Ba deviated significantly from the ratio 0.764 ± 0.005 of gamma-ray emission probabilities of ^133^Ba (62.05 ± 0.19) % and ^131^I (81.2 ± 0.5) % [20, 21] and was observed to be highly dependent on the setup used. Although the deviation was smaller for setups including a Monte Carlo-based scatter correction, a site-specific cross-calibration procedure is recommended. Once a setup-specific cross-calibration line is in place, however, quality control measurements such as stability measurements can be performed based on solid ^133^Ba sources only. This solves the radiation protection problem inherent in the preparation of phantoms with liquid activity solutions and reduces the measurement uncertainties in the activity.

One thing that should be noted is that the uncertainty calculation for the pseudo-ICF values includes the counts inside the cylinders taken from the reconstructed attenuation-corrected images. Given the small number of total counts in the smallest cylinder, this might lead to a considerable overestimation of the counts and, as a result, to an underestimation of the uncertainties especially for the small cylinders. Similarly, the assumption that the counts within the considered volumes can be approximated by Poisson statistics might lead to an underestimation of the uncertainty in the number of counts, in particular for the smaller cylinder inserts. However, our study design required on-site reconstruction (including attenuation correction) and analysis by each participating site before results were collected for evaluation, which eliminated the possibility of a projection-based calculation of the uncertainty. While not investigated in this work, a more detailed analysis of the resulting attenuation-dependent underestimation of the uncertainty could become part of a follow-up study.

Although a more detailed analysis of a potential partial volume correction would have been interesting, it was not possible on the basis of the available data. For example, the enlarged VOIs (i.e. including counts lost due to spill-out) proposed in the SOP for that very purpose were not available for all sites. The available VOIs had the same (nominal cylinder) volume, but had been drawn using different VOI drawing methods, the choice of which (exact vs. threshold-based) can lead to additional non-negligible differences in pseudo-ICF, and prevented a systematic analysis of partial volume errors. This shortcoming is inherent in the study design and needs to be improved in future studies.

Other radionuclides with similar main energies could potentially be better suited as an analogue for ^131^I for imaging than ^133^Ba. As an example, ^113^Sn has only one major gamma emission line at (391.698 ± 0.007) keV (emission probability: (64.97 ± 0.17) %). However, due to the shorter half-life of 115.1 days, this radionuclide is useful for system calibration, but limited as a source for system stability assessments and long-term quality control compared to a long-lived source such as ^133^Ba with a half-life of 10.5 years. A potential solution could be to calibrate a system with a traceable ^113^Sn source and use a cross-calibrated ^133^Ba source for system stability assessments. Although ^133^Ba has been shown not to be a good substitute for ^131^I in radionuclide calibrator measurements, this knowledge is of minor importance in the cross-calibration of SPECT/CT systems, since solid sources are typically produced or supplied with traceable activities and consequently do not need to be measured in the radionuclide calibrator.

Although this exercise was affected by challenges associated with the logistics of the source transportation, the long half-life of ^133^Ba mitigated any issues arising from this. While the inland transport between the UK sites was rather straightforward, transportation between different countries as UN2910 radioactive material excepted package turned out to be difficult due to differences in the regulations of participating countries. In addition, source transport via air proved to be not firmly plannable as the decision whether to carry the sources is the sole responsibility of the respective aircraft pilot. Consequently, in some instances, the sources had to be spontaneously transported by road between participating countries such as from Germany to the UK or, even more costly, from Sweden to Italy. Therefore, the logistics and potentially high transportation costs must be taken into full consideration when planning an international multi-centre study to avoid any major delays.

This study demonstrated that ^133^Ba can be used as a surrogate for liquid ^131^I for quality control and quantitative SPECT/CT. The use of ^133^Ba greatly reduces the time needed for preparation of quality control measurements (no preparation of stock solution or phantom filling) and can mitigate potential errors in the source preparation. The use of solid ^133^Ba sources also provides benefits in reduced radiation exposure and volatility.

## Conclusion

This study presented the results of an international comparison exercise on the feasibility of a reliable calibration of SPECT/CT systems for quantitative ^131^I imaging based on a set of ^133^Ba surrogate sources following a harmonised protocol. Based on the results obtained from eight imaging centres, a site-specific cross-calibration is recommended to minimise the differences between ^131^I and ^133^Ba. In multi-centre setups, care should be taken that different sites follow the same VOI drawing technique, as this had a major impact on the cross-calibration especially for the smaller sources. In summary, the use of traceable ^133^Ba sources has the potential to reduce the inherent problems with on-site activity measurement and phantom preparation with ^131^I.

### Supplementary Information

**Additional file 1.** ^133^Ba-cylinder pseudo-ICFs for the individual CEA and CMI sources and the combined average, with the corresponding uncertainties.

## Data Availability

The datasets used and/or analysed during the current study are available from the corresponding author on reasonable request.
