# Supplementary material for: On the use of solid 133Ba sources as surrogate for liquid 131I in SPECT/CT calibration: a European multi-centre evaluation
Source: EJNMMI Phys. 2023 Nov 23;10:73. doi: 10.1186/s40658-023-00582-3 (PMC10665282; doi:10.1186/s40658-023-00582-3)
Supplement: Supplementary file 1 — Additional file 1. 133Ba-cylinder pseudo-ICFs for the individual CEA and CMI sources and the combined average, with the corresponding uncertainties. [file 40658_2023_582_MOESM1_ESM.pdf]

On the Use of Solid ^{133}Ba Sources as Surrogate for Liquid ^{131}I in SPECT/CT Calibration – a European Multi-Centre Evaluation – Supplementary Data

Authors:

Tran-Gia, Johannes[1]*, Denis-Bacelar, Ana M[2], Ferreira, Kelley M[2], Robinson, Andrew P[2], Bobin, Christophe[3], Bonney, Lara M[4], Calvert, Nicholas[5], Collins, Sean M[2,6], Fenwick, Andrew J[2], Finocchiaro, Domenico[7], Fioroni, Federica[7], Giannopoulou, Katerina[8], Grassi, Elisa[7], Heetun, Warda[2], Jewitt, Stephanie J[4], Kotzasarlidou, Maria[8], Ljungberg, Michael[9], Lourenço, Valérie[3], McGowan, Daniel R[4,10], Mewburn-Crook, Jamie[2], Sabot, Benoit[3], Scuffham, James[11], Sjögreen Gleisner, Katarina[9], Solc, Jaroslav[12], Thiam, Cheick[3], Tipping, Jill[4], Wevrett, Jill[11], The MRTDosimetry Collaboration, Lassmann, Michael[1]

*Corresponding author

Affiliations:

1 Department of Nuclear Medicine, University Hospital Würzburg, Würzburg, Germany
2 National Physical Laboratory, Hampton Road, Teddington, UK
3 Université Paris-Saclay, CEA, List, Laboratoire national Henri Becquerel (LNE-LNHB), F-91120 Palaiseau, France
4 Department of Medical Physics and Clinical Engineering, Churchill Hospital, Oxford University Hospitals NHS Foundation Trust, Oxford, UK
5 Christie Medical Physics and Engineering (CMPE), The Christie NHS Foundation Trust, Manchester, UK
6 School of Mathematics and Physics, University of Surrey, Guildford, UK
7 Medical Physics Unit, Azienda USL-IRCCS di Reggio Emilia, Reggio Emilia, Italy
8 Nuclear Medicine Department, "THEAGENIO" Anticancer Hospital, Thessaloniki, Greece
9 Medical Radiation Physics, Lund, Lund University, Lund, Sweden.
10 Department of Oncology, University of Oxford, Oxford, UK.
11 Royal Surrey County Hospital, Royal Surrey NHS Foundation Trust, Guildford, UK
12 Czech Metrology Institute, Okruzni 31, 638 00, Brno, Czech Republic

Table 1. ^{133}Ba-cylinder pseudo-ICFs for the individual CEA and CMI sources and the combined average, with the corresponding uncertainties.

	**C1 7.5 mm**			**C2 15 mm**			**C3 30 mm**			**C4 60 mm**		
	**CEA**	**CMI**	**Average**	**CEA**	**CMI**	**Average**	**CEA**	**CMI**	**Average**	**CEA**	**CMI**	**Average**
**S1**	7.1 (0.1)	7.3 (0.3)	7.2 (0.4)	20.7 (0.4)	21.5 (0.2)	21.1 (0.3)	28.9 (0.3)	28.8 (0.4)	28.8 (0.1)	32.3 (0.5)	32.8 (0.3)	32.6 (0.4)
**S2a**	27.8 (0.4)	33 (1)	30 (1)	80 (1)	78.0 (0.5)	78.9 (0.9)	96 (1)	96 (1)	96 (3)	108 (1)	111.9 (0.9)	110 (2)
**S2b**	14.1 (0.2)	15.7 (0.6)	14.9 (0.6)	43.0 (0.6)	45.6 (0.3)	44.3 (0.5)	48.2 (0.6)	51.0 (0.6)	49.6 (0.8)	49.7 (1)	53 (2)	52 (2)
**S2c**	7.0 (0.1)	7.0 (0.3)	7.0 (0.4)	20.2 (0.4)	20.8 (0.2)	20.5 (0.3)	28.2 (0.3)	27.8 (0.4)	28.0 (0.1)	31.6 (0.4)	31.8 (0.3)	31.7 (0.3)
**S2d**	7.6 (0.2)	6.2 (0.3)	6.9 (0.4)	23.9 (0.4)	18 (0.2)	21.0 (0.2)	27.8 (0.3)	22.3 (0.3)	25.0 (0.7)	30 (3)	25 (3)	27 (2)
**S3**	5.0 (0.2)	4.2 (0.3)	4.6 (0.3)	21.8 (0.4)	22.3 (0.3)	22.0 (0.3)	27.8 (0.3)	28.8 (0.3)	28.3 (0.4)	30.1 (0.3)	31.6 (0.5)	30.9 (0.8)
**S4**	25.9 (0.4)	29 (1)	27 (1)	76 (1)	85.0 (0.4)	81 (1)	100 (1)	105 (1)	103 (1)	107 (4)	115 (3)	111 (4)
**S5**	31.2 (0.5)	31 (1)	31 (1)	79 (1)	79.5 (0.5)	79.0 (0.9)	102 (1)	98 (1)	100.1 (0.3)	110.7 (0.8)	108 (2)	109 (2)
**S6**	8.8 (0.2)	7.2 (0.3)	8 (0.4)	22.7 (0.4)	23.8 (0.2)	23.2 (0.3)	27.2 (0.3)	27.1 (0.3)	27.2 (0.8)	27.7 (0.6)	28.5 (0.2)	28.1 (0.4)
**S7**	7.7 (0.2)	9.1 (0.3)	8.4 (0.4)	22.6 (0.4)	24.2 (0.2)	23.4 (0.3)	29.3 (0.3)	30.9 (0.4)	30.1 (0.7)	32.1 (0.8)	33.2 (0.8)	32.6 (0.6)
**S8**	13 (0.2)	11.1 (0.5)	12.1 (0.6)	35.4 (0.7)	31.2 (0.2)	33.3 (0.4)	47.2 (0.5)	40.7 (0.5)	44 (1)	51 (2)	46 (3)	48 (3)

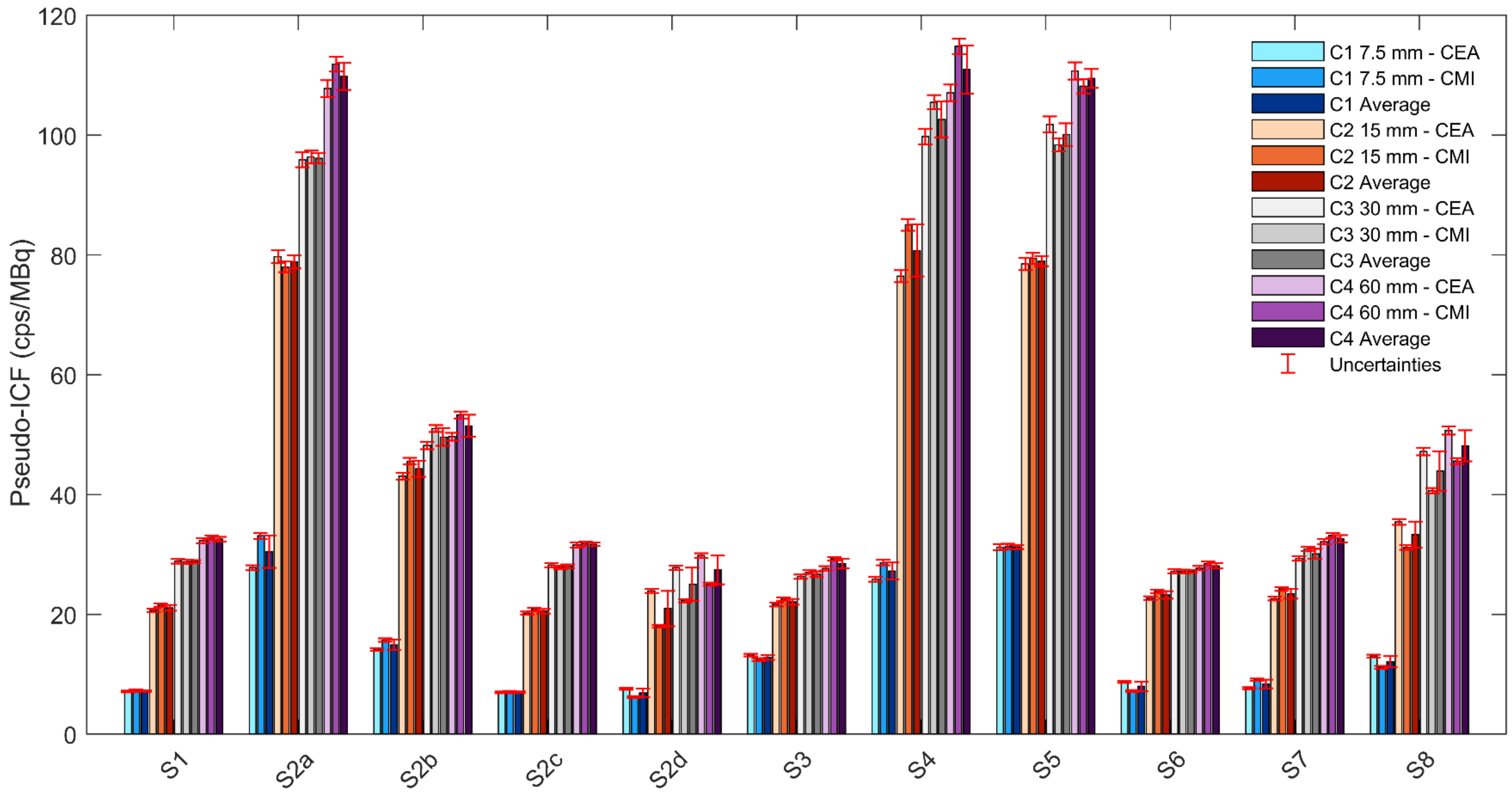

Figure 1. ^{133}Ba-cylinder pseudo-ICFs for the individual CEA and CMI sources and the combined average, with the corresponding uncertainties.
